# How Mandarin–English bilinguals interpret *qián*/*forward*: impact of language proficiencies on retrieval of temporal concepts

**DOI:** 10.3389/fpsyg.2024.1370605

**Published:** 2024-05-07

**Authors:** Tengji Yang, Ying Yang

**Affiliations:** ^1^Foreign Studies College, Hunan Normal University, Changsha, China; ^2^Department of Psychology, School of Education Science, Hunan Normal University, Changsha, China; ^3^Cognition and Human Behavior Key Laboratory of Hunan Province, Hunan Normal University, Changsha, China

**Keywords:** metaphor, bilingual, language proficiency, temporal concepts, retrieval

## Abstract

Mandarin *qián* and English *forward* are semantically equivalent in the domain of Space, but could be semantically opposite in the domain of Time. In other words, equivalent spatial lexical items could convey opposite temporal concepts. What temporal concepts conveyed by *qián* and *forward* would be retrieved by Mandarin–English (M–E) bilinguals with different language proficiencies? Drawing a sample from college students in Mainland China, this study examines how L1 and L2 proficiencies would affect M–E bilinguals' retrieval of temporal concepts by examining their interpretation of the Mandarin temporal metaphor of *qián* and the English temporal metaphors of *forward*. The results show that L1 temporal concepts would be retrieved more frequently than L2 temporal concepts regardless of the testing languages, that L1 and L2 proficiencies were not predictors for the way of interpretation, and that the higher L2 proficiency group could retrieve temporal concepts in line with the testing languages with higher accuracy than the lower L2 proficiency group. The findings suggest that bilinguals with higher L2 proficiency may be able to represent temporal concepts with language tags or may have an attentional and/or inhibitory control advantage.

## 1 Introduction

Bilinguals know more than one language, and how bilinguals represent, access, and retrieve concepts from their two languages is of interest to many studies. Many lexicon models have been proposed to answer this question. Most lexical models have distinguished two levels, one being the lexical level (i.e., the surface level) and the other being the conceptual level (i.e., the deep level) (Sánchez-Casas and García-Albea, 2005). While some propose that bilinguals would have one common inventory of concepts for L1 and L2 (e.g., Potter et al., 1984; Kroll and Stewart, 1994), others believe that there would be two inventories of concepts for L1 and L2, although there should be some overlap between the two inventories (e.g., Dong et al., 2005; Pavlenko, 2009; Li, 2017; Li, 2019). Many factors would influence the degree of interconnection between concepts from the two languages. Some factors are related to bilinguals, such as L2 proficiency, age of acquisition (AoA), L2 experience, language learning context, etc. (Lambert, 1969; Kroll and Stewart, 1994; De Groot and Poot, 1997; Li, 2019); some factors are related to languages, such as cognate status, grammatical category of the word associated with the concept, concreteness, etc. (De Groot, 2001; Sánchez-Casas and García-Albea, 2005; Mätzig et al., 2009). According to previous studies (De Groot, 1995; Van Hell and De Groot, 1998), abstract concepts are represented and accessed differently from concrete concepts, so that abstract concepts are retrieved more slowly. By drawing a sample from a homogeneous Mandarin–English (M–E) bilingual population who were college students living in China since birth, this study would explore the characteristics of M–E bilinguals' retrieval of abstract temporal concepts via concrete spatial lexical items by focusing on the effect of language proficiencies.

According to the Conceptual Metaphor Theory (Lakoff and Johnson, 1980, 1999; Lakoff, 1993), concrete spatial concepts can be used to construct abstract temporal concepts, which correspond to spatio-temporal conceptual metaphors in people's minds and are reflected in spatio-temporal metaphorical expressions in people's languages. Previous studies have demonstrated the psychological reality of spatio-temporal metaphors in people's minds (e.g., Boroditsky, 2000; Boroditsky and Ramscar, 2002; Evans, 2004; Casasanto and Boroditsky, 2008; Ishihara et al., 2008; Li and Cao, 2019) and have shown that spatio-temporal metaphorical expressions are widespread in many languages (e.g., Haspelmath, 1997; Moore, 2011; Radden, 2011; Yu, 2012; Duffy and Feist, 2023, p. 50–81). Different languages would explore different spatial lexical items to convey the same temporal concept (e.g., Boroditsky and Gaby, 2010; Gaby, 2012), or the equivalent spatial lexical items would be used to convey different temporal concepts (e.g., Chen, 2007, p. 137–139; Lin and Li, 2018; Liu et al., 2018; He, 2019, p. 118–124). When Mandarin and English spatio-temporal metaphorical expressions were contrasted, the differences were mainly reflected in the following two aspects. First, there are many more vertical spatial lexical items that convey temporal concepts in Mandarin (Scott, 1989; Yu, 1998, p. 110–112; Lan, 2002; Radden, 2011; Lin, 2015, p. 38–39; Wei, 2019; Xiao, 2019, p. 106–148). Second, when the temporal concepts of *Earlier/Past* need to be expressed by horizontal spatial lexical items, *qián/wǎng-qián* (literally “front/forward”) would be used in Mandarin, while *back/backward* would be used in English (Liu, 1993; Chen, 2007, p. 137–139, 2021; Zhang and Luo, 2007; Li and Zhang, 2017; Lin and Li, 2018; Liu et al., 2018; He, 2019, p. 118–124; Chen and Zhang, 2021). In other words, although Mandarin *qián/wǎng-qián* and English *front*/*forward* are equivalent spatial lexical items, they could denote opposite temporal concepts in the Time domain. Therefore, as Chen (2021) pointed out, *front/forward* in many English temporal expressions is translated into Mandarin as *hòu/wǎng-hòu* (literally “back/backward”), and *back/backward* in many English temporal expressions is translated into Mandarin as *qián/wǎng-qián* (literally “front/forward”).

Since spatio-temporal metaphorical expressions are closely related to people's mental concepts, how would M–E bilinguals accommodate differences in spatio-temporal metaphorical expressions between two languages and represent temporal concepts in their minds? Some studies have compared M–E bilinguals with Mandarin (L1) monolinguals to investigate whether learning English (L2) would make M–E bilinguals' retrieval and representation of temporal concepts different from that of L1 monolinguals (Lai and Boroditsky, 2013; Yang and Wen, 2014; Zhang et al., 2016; Li and Zhang, 2019; Yang et al., 2022). Results show that if temporal concepts were accessed via vertical spatial cues or lexical items, the representation of temporal concepts by M–E bilinguals would not differ from that of L1 monolinguals (Yang and Wen, 2014; Zhang et al., 2016; Yang et al., 2022). However, if their temporal concepts were accessed via horizontal spatial lexical items, the retrieval of temporal concepts by M–E bilinguals would differ from that of L1 monolinguals (Lai and Boroditsky, 2013; Li and Zhang, 2019). We believe that the difference in research findings could be explained by semantic consistency and semantic discrepancy. Semantic consistency is demonstrated by Mandarin and English vertical spatio-temporal metaphors (i.e., vertical spatial lexical items), as Mandarin *shàng* (literally “up”) and English *up* denote the same temporal concept of *Earlier*, and Mandarin *xià* (literally “down”) and English *down* denote the same temporal concept of *Later* (Yu, 1998, p. 110–112). Therefore, M–E bilinguals' exposure to L2 vertical spatio-temporal metaphors would not affect their temporal concepts. In contrast, a semantic discrepancy is shown by Mandarin and English horizontal spatio-temporal metaphors, as Mandarin *qián*/*wǎng-qián* (literally “front/forward”) means *Earlier/Past*, while English *front*/*forward* means the opposite temporal concept of *Future*/*Later* (e.g., Liu et al., 2018; Chen and Zhang, 2021). When M–E bilinguals learn L2, their exposure to L2 horizontal spatio-temporal metaphors may influence M–E bilinguals' temporal concepts, resulting in a different response from that of L1 monolinguals. Therefore, M–E bilinguals' interpretation of *qián-forward* would provide us with an opportunity to understand their retrieval and representation of temporal concepts. By examining M–E bilinguals' interpretation of *qián*-*forward*, we can investigate the extent to which the retrieved temporal concepts would be in line with L1 spatio-temporal metaphors (i.e., retrieving the concepts of *Earlier/Past*) or L2 spatio-temporal metaphors (i.e., retrieving the concepts of *Future*/*Later*) via equivalent horizontal lexical items. In this way, we can examine the influence of language proficiencies on M–E bilinguals' representation of temporal concepts.

Previous studies have investigated M–E bilinguals' representation and retrieval of temporal concepts via horizontal spatial lexical items. Lai and Boroditsky's (2013) study showed that M–E bilinguals' representation of temporal concepts would differ from that of L1 monolinguals because these two groups interpreted Mandarin *qián* differently, and that M–E bilinguals' representation of temporal concepts would differ from that of L2 monolinguals because these two groups interpreted English *forward* differently. Furthermore, according to Lai and Boroditsky (2013), L1 and L2 proficiencies could predict the way people interpret *qián-forward*. Since the above result was based on comparing M–E bilinguals with L1 and L2 monolinguals, the predictive effect of L1 and L2 proficiencies could be verified by focusing on M–E bilinguals with different L1 and L2 proficiencies. In addition, Li and Zhang (2019) compared M–E bilinguals with high L2 proficiency (i.e., prost-graduates in English majors) with L1 monolinguals and L2 monolinguals, and found that M–E bilinguals' interpretation was different from L1 and L2 monolinguals in the low cognitive load task, but similar to L1 monolinguals in the high cognitive load task. The above two studies were focused on comparing bilinguals with monolinguals. However, Rothman et al. (2023) argued that such a comparison would have masked confounds, thus unnecessarily compromising a general understanding of bilingual data and limiting the set of questions to be asked in the field of bilingualism. In the above two studies of M–E bilinguals, the possible interaction between accessing languages and L2 proficiency was ruled out. L1 was used as the only accessing language when comparing M–E bilinguals with L1 monolinguals in Lai and Boroditsky's (2013) study, and different accessing languages were given only to M–E bilinguals with high L2 proficiency in Li and Zhang's (2019) study. The above designs made it impossible to investigate the extent to which M–E bilinguals with different proficiencies would respond differently/similarly to the two testing languages. As shown by Fuhrman et al. (2011), the testing languages would influence the way M–E bilinguals organize time. Conversely, according to Li and Zhang (2019), testing languages would not affect the temporal concept retrieval of M–E bilinguals with advanced L2 proficiency. This may suggest that M–E bilinguals with different language proficiencies would respond differently to the two testing languages.

The present study aimed to investigate how L1 and L2 proficiencies would affect M–E bilinguals' retrieval of temporal concepts by answering the following two questions. First, would L1 and L2 proficiencies predict how M–E bilinguals retrieve temporal concepts? Second, would M–E bilinguals with different language proficiencies respond differently to the two testing languages? In Mainland China, many people are M–E bilinguals who acquire L1 from infancy and learn L2 at the age of eight or nine in primary school. L1 and L2 tests are required in college entrance exams. According to Lambert's (1969) definition, most college students in Mainland China are coordinated bilinguals who learn L1 and L2 in succession.^1^ Their population is large, as there were 18,931,044 four-year college students in 2021,^2^ and many of them were non-English majors. Their L2 learning contexts are limited, as most non-English majors learn the L2 in an environment with native L1 speakers. Their L2 learning time is limited, because most non-English majors learn the L2 exclusively in English classes. At the same time, their proficiency levels vary, with some scoring more than 140 points on the L1 and L2 tests of the college entrance exam (the full score is 150 points), and some scoring less than 50 points on the same exam. Their interpretation of *qián-forward* would help us investigate the predictive effect of L1 and L2 proficiencies on M–E bilinguals' temporal concept retrieval, and the extent to which M–E bilinguals with different language proficiencies would respond differently/similarly to the two testing languages.

In different models of the bilingual lexicon, L1 and proficiencies played an important role. Since most bilinguals have unbalanced L1 and L2 proficiencies, the unbalanced feature was represented in different ways by different models. Some models used two lexicons (i.e., inventories of lexical items) of different sizes to capture the unbalanced feature, with the larger L1 lexicon representing higher L1 proficiency and the smaller L2 lexicon representing lower L2 proficiency (e.g., Potter et al., 1984; Kroll and Stewart, 1994; Dong et al., 2005; Pavlenko, 2009). Some models used different access links between the lexical and the conceptual levels to represent unbalanced proficiencies, with L1 access links being stronger than L2 access links (e.g., Kroll and Stewart, 1994; Dong et al., 2005; Li, 2017). Some models used concept inventories of different sizes to represent the unbalanced proficiencies, with the L1 concept inventory being larger than the L2 concept inventory (e.g., Pavlenko, 2009; Li, 2017; Li, 2019). As described in the above models, bilinguals' L1 lexicon would be larger than the L2 lexicon, and/or the access links between L1 lexical items and L1 concepts would be stronger, and/or the L1 concept inventory would be larger than the L2 concept inventory. Therefore, L1 concepts would be retrieved more frequently than L2 concepts. Based on the above lexicon models and discussion, Hypothesis 1a is stated as follows:

*Hypothesis 1a: Regardless of the testing languages, participants would give the interpretation in line with L1 metaphors (i.e., the interpretation of Past/Earlier) with higher frequency than the interpretation in line with L2 metaphors (i.e., the interpretation of Future/Later)*.

Lai and Boroditsky (2013) showed that the L1 and L2 proficiencies of M–E bilinguals would predict the way in which M–E bilinguals retrieved temporal concepts. Specifically, the L1 proficiency was the predictor of retrieving L1 temporal concepts, and L2 proficiency was the predictor of retrieving L2 temporal concepts. According to lexicon models, with the improvement of L2 proficiency and the increase of L2 experience, bilinguals' L2 concept inventory would be expanded (e.g., Li, 2017; Li, 2019), and/or the access links between L2 lexical items and L2 concepts would become stronger (e.g., Kroll and Stewart, 1994; Dong et al., 2005; Li, 2017). Presumably, bilinguals with high L2 proficiency would have a larger L2 concept inventory than bilinguals with low L2 proficiency, so that bilinguals with high L2 proficiency would process L2 concepts faster or more accurately or retrieve L2 concepts more frequently than bilinguals with low L2 proficiency. The role of the L2 proficiency predicted by the above models has been confirmed by several studies. L2 proficiency was found to be positively related to the frequency of retrieving temporal concepts in line with L2 metaphors (Lai and Boroditsky, 2013), at least in the cognitively unloaded condition (Li and Zhang, 2019). Since the samples of the two studies were not non-English majors living in Mainland China, the role of L1 and L2 proficiencies could be further tested in this group of bilinguals. Hypothesis 1b is stated as follows:

*Hypothesis 1b: Higher L1 proficiency would be related to a higher frequency of interpretation in line with L1 metaphors (i.e., the interpretation of Past/Earlier); higher L2 proficiency would be related to a higher frequency of interpretation in line with L2 metaphors (i.e., the interpretation of Future/Later)*.

According to the Inhibitory Control Model of bilingualism, the supervisory attentional system (SAS) monitors the activation of the testing language and its concepts, as well as the inhibition of the non-testing language and its concepts (Green, 1998). According to the adaptive control hypothesis (Green and Abutalebi, 2013), eight control processes (e.g., goal maintenance, conflict monitoring, interference suppression, etc.) would be involved in bilingual interactional contexts. With constant monitoring and control, bilinguals would have cognitive advantages (e.g., Ware et al., 2020; Tao et al., 2021), and higher levels of bilingualism were partially associated with improved attentional function (Privitera et al., 2023). Furthermore, Li and Zhang (2019) showed that L2 proficient M–E bilinguals' retrieval of L1 temporal concepts was related to the cognitive load of the task, in that L1 temporal concepts were retrieved more frequently via L2 spatial lexical items in the cognitively loaded condition than in the cognitively unloaded condition. The result would suggest that cognitive resources were allocated to the cognitively loaded task, making it difficult for participants to control L1 transfer when tested in L2 (Li and Zhang, 2019). Based on the above models and studies, it could be hypothesized that L2 proficiency would be positively related to cognitive advantage, which would predict better control over language transfer and interference. Thus, Hypothesis 2 is stated as follows:

*Hypothesis 2: Participants with higher L2 proficiency would retrieve temporal concepts in line with the testing languages with higher accuracy than participants with lower L2 proficiency*.

By testing the above hypotheses, the present study would answer how L1 and L2 proficiencies would affect M–E bilinguals' retrieval of temporal concepts.

## 2 Methods

### 2.1 Participants

Power analysis indicated that a sample size of 166 participants could yield an estimated medium effect size (G^*^Power 3.1, power = 0.95, effect size *w* = 0.28, α = 0.05, between-subjects design) (Faul et al., 2007). To optimize the test results, 384 participants were recruited from a public comprehensive university in Changsha, a central city in Mainland China. Participants (*n* = 384, 282 females; *M*_*age*_ = 18.97 years, *SD*_*age*_ = 0.66 years) were all native Mandarin (L1) speakers and non-English majors. For most participants, English (L2) classes were the only L2 immersion time (e.g., 135 min per week for first-year students, 67.5 min per week for second-year students, and none for third- or fourth-year students). They shared similar language learning contexts in which L2 classes were delivered by native L1 speakers and interactive practice was conducted among native L1-speaking classmates. Participants were given small gifts at the end of the study, and informed written consent was obtained from them. The study was approved by the Scientific Research Ethics Committee of Hunan Normal University, China (2024-352). Participants were randomly assigned to either the L1 condition (i.e., Mandarin; *n* = 192, 72.92% female) or the L2 condition (i.e., English; *n* = 192, 74.35% female) in equal proportions based on self-reported gender.

### 2.2 Materials

#### 2.2.1 Language background questionnaire

Language background information was collected using a questionnaire written in Mandarin. Participants were asked to report their Mandarin proficiency on a five-point scale and their English proficiency on another five-point scale of the same design (with 5 being very proficient and 1 being very non-proficient). They were also asked to report their highest scores on the College English Test-4 (CET-4). They were asked to list languages they use other than Mandarin and English and to indicate their proficiency by using a 5-point scale.

Self-reported Mandarin proficiency was used to measure L1 proficiency in accordance with previous studies (Lai and Boroditsky, 2013). Self-reported English proficiency was used to subjectively measure L2 proficiency. CET-4 scores were used to objectively measure L2 proficiency. College English Tests (CETs) are composed of writing, listening comprehension, reading, and translation from Mandarin to English and are widely and exclusively taken by non-English majors in Mainland China. Many universities in China have made passing the CET-4 a requirement for graduation. The total score for the CET-4 is 710, and scores of 425 and above are considered passing. As some participants may use languages other than Mandarin and English, information on this aspect was also collected.

#### 2.2.2 Interpretation testing questionnaire

Following previous studies that used the *forward*-interpretation question to retrieve temporal concepts (McGlone and Harding, 1998; Boroditsky and Ramscar, 2002; Kranjec, 2006; Kranjec and McDonough, 2011; Lai and Boroditsky, 2013; Duffy and Feist, 2014; Li and Zhang, 2019), this study used Mandarin (in the L1 condition) and English (in the L2 condition) questionnaires that were semantically equivalent. Three multiple-choice questions about Time were included, as shown in Tables 1, 2.

**Table 1:** Time questions in the English version of the questionnaire.

(1) Mark was born in September, and Jane was born in December of the same year. Who is older, Mark or Jane?		
** A. Mark**	** B. Jane**	**C. Don't know**
(2) Mark was born on October 20, and Jane was born on October 10 of the same year. How many days are there between their birthdays?		
A. 20 days	B. 10 days	C. Don't know
(3) Suppose the clock says it is 10 o'clock. You need to move it 1 h forward. What time will it be adjusted to?		
A. 9 o'clock	B. 11 o' clock	C. Don't know

**Table 2:** Time questions in the Mandarin version of the questionnaire.

(1)	小明	生	于	9月,	小红	生	于	同年		12月。
	xiao-ming	sheng	yu	jiu-yue,	xiao-hong	sheng	yu	tong	nian	shi-er-yue.
	Xiaoming	born	in	September	Xiaohong	born	in	same	year	December.

	请问	小明	和	小红	谁	大？
	qing wen	xiao-ming	he	xiao-hong	shui	da?
	please ask	Xiaoming	and	Xiaohong	who	big?
	A.	小明	B.	小红	C.	不	知道
		xiao-ming		xiao-hong		bu	zhi-dao
		Xiaoming		Xiaohong		not	know
(2)	小明	生	于	10月	20日,	小红	生	于	同年	10月
	xiao-ming	sheng	yu	shi-yue	er-shi ri,	xiao-hong	sheng	yu	tong nian	shi-yue
	Xiaoming	born	in	October	20th day,	Xiaohong	born	in	same year	October
	10日。	请	问	他们	生日	差	几	天？
	shi-ri.	qing	wen	ta-men	sheng-ri	cha	ji	tian?
	10th day.	please	ask	they	birthday	differ	how many	days?
	A.	20天	B.	10天 (10 days)	C.	不知道	
		er-shi tian		shi tian		bu	zhi-dao
		20 day		10 day		not	know
(3)	假设	时钟	显示	10点。	请	你	把	它	往前	调	1
	jia-she	shi-zhong	xian-shi	shi-dian.	qing	ni	ba	ta	wang-qian	tiao	yi
	Suppose	clock	show	10 o'clock.	Please	you	make	it	forward	move	one
	小时。	请	问	调	好	的	时钟	显示	几	点？
	xiao-shi.	qing	wen	tiao	hao	de	shi-zhong	xian-shi	ji	dian?
	hour.	please	ask	adjust	good	particle	clock	show	how many	clock?
	A.	9点	B.	11点	C.	不	知道
		jiu dian		shi-yi dian		bu	zhi-dao
		9 o'clock		11 o'clock		not	know

The first two Time questions were designed to help participants access the concept of Time through the testing language, and effective answers to these two Time questions were regarded as understanding questions in this questionnaire. For the first Time question, choosing A was taken as the correct answer, choosing B was taken as the incorrect answer, and other responses were taken as ineffective answers (i.e., choosing C, giving more than one choice, or giving no choice). For the second Time question, choosing B was taken as the correct answer, choosing A was taken as the incorrect answer, and other responses were taken as ineffective answers. Both correct and incorrect answers were taken as effective answers.

The third Time question was the target question. The target question in this study was adapted from the clock question used by Lai and Boroditsky (2013). The L2 version of the questionnaire read, “Suppose the clock says it is 10 o'clock. You need to move it 1 h forward. What time will it be adjusted to?” Choosing A (i.e., 9 o' clock) indicated that *forward* was interpreted as *Earlier*/*Past*, and this interpretation was regarded as retrieving L1 temporal concepts, which was in line with the L1 metaphor. In Mandarin, the morpheme of *qián* (“front/forward”) is very often used to mean *Earlier*/*Past* (e.g., Alverson, 1994; Yu, 1998; Cai, 2012; Liu et al., 2018; Chen, 2021; Chen and Zhang, 2021), so interpreting *qián* as *Earlier*/*Past* is the unmarked situation (i.e., the normal situation) (Shen, 2015; Wang, 2016). Conversely, choosing B (i.e., 11 o' clock) indicated that *forward* was interpreted as *Future*/*Later*, and this interpretation was regarded as the retrieval of L2 temporal concepts, which was in line with the L2 metaphor. In Mandarin, *qián* (“front/forward”) is rarely used to mean *Future*/*Later* (Shen, 2015; Liu et al., 2018). Moreover, in English, *back* is used to mean *Earlier*/*Past* at a much higher frequency than *forward*, so that *back* is translated into Mandarin as *qián* in many temporal expressions (Chen, 2021). Other responses to the third question were regarded as ineffective responses (i.e., choosing C, giving more than one choice, or giving no choice). Similar coding was applied to responses to the L1 version of the questionnaire.

There is one point that needs more explanation. Studies (Moore, 2006, 2011; Núñez and Sweeter, 2006; Núñez et al., 2006; Yu, 2012; Núñez and Cooperrider, 2013; Xiao et al., 2018) show that there would be at least two sets of temporal orientations (i.e., two temporal frames of reference). One set would be ego-free, including *Earlier-Later* orientations, and the other set would be ego-based, including *Past-Future* orientations. Both *Later* and *Future* would be oriented in line with the arrow of time, and both *Earlier and Past* would be oriented against the arrow of time. As Moore (2006) pointed out, the difference between the two sets would not depend on linguistic cues such as deictic expressions (e.g., tense), but on the perspectives of the conceptualizers (i.e., speakers or addressees). In other words, the difference between *Earlier* and *Past* depended on the perspective of the speaker and/or addressee, not on linguistic cues, and the same is true for the difference between *Later* and *Future*. Since the perspective of the participants was not checked or controlled, we believe that the *qián*-*forward* question used in this study and other similar studies would not distinguish *Earlier* from *Past*, nor would it distinguish *Future* from *Later*. Therefore, effective answers to the third Time question (i.e., choosing A or B) were coded as *Earlier*/*Past* vs. *Future*/*Later*.

### 2.3 Procedure

Prior to the study, participants were told that the study was designed to test their language proficiency and that they would receive small gifts if they completed the questionnaires. Participants were then randomly assigned to the L1 or L2 condition. In both conditions, the Language Background Questionnaire was given first, followed by the Interpretation Testing Questionnaire. The Language Background Questionnaires were the same for both conditions, with questions given in L1. The Interpretation Testing Questionnaires were different for the two conditions. In the L1 condition, the L1 version of the interpretation testing questionnaires was given. In the L2 condition, the L2 version of the interpretation testing questionnaires was given. In this process, no discussion was allowed, and no time limit was set for answering the questions. Then, the researchers collected the questionnaires and the participants' informed written consent by explaining the true purpose of the study and giving gifts. Each participant took ~10 min to complete the task.

### 2.4 Data analysis

Answers from 384 participants were coded using SPSS 27. First, data from seven participants who did not give an accurate CET-4 score (i.e., only gave “400+”) were excluded. Data from eight participants who gave ineffective answers to any of the three Time questions were excluded. No participants gave incorrect answers to both the first and second Time questions (although some gave an incorrect answer to either the first or second Time question), indicating that they could understand the interpretation task. Data from five participants whose self-reported L1 proficiency was less than three points were excluded (i.e., Mandarin may not be their L1). Data from two participants who reported higher proficiency in the third language than in English were excluded (i.e., English may not be their L2). Participants who reported dialects (e.g., Changsha dialect) were not coded as using L3 because dialects are very similar to Mandarin in phonological, morphological and syntactic terms and are considered to be Mandarin (e.g., Lu, 2013). Therefore, data from 362 participants were analyzed. Their demographic information, language background, and response data are shown in Table 3.

**Table 3:** Demographic, language background, and response data.

	***M***	**SD**	**Range**
Age (years)	18.98	0.653	18–21
L1 proficiency (1–5 point)	3.965	0.698	3–5
L2 proficiency SUB (1–5 point)	2.728	0.686	1–5
L2 proficiency OBJ (0–710 point)	492.9	69.894	334–646
	**Frequency**	**Percentage**	
Sex (female)	269	74.3%	
L3	16	7.7%	
Testing language (L1)	184	50.8%	
Time Question 1 (correct answer)	334	92.3%	
Time Question 2 (correct answer)	359	99.2%	
Time Question 3 (*Past/Earlier* interpretation)	292	80.7%	

The effects of L1 proficiency and L2 proficiency were tested. The effect of L1 proficiency was tested using logistic regression. The effect of L2 proficiency was tested using L2 Proficiency OBJ (i.e., CET scores). L2 proficiency SUB (i.e., self-reported L2 proficiency) was not tested because Zhou and Privitera (2024) found that subjective and objective proficiency measures would not lead to a difference. First, the variable of L2 proficiency was used as a scale variable, so its effect was tested via logistic regression. Second, as the sample was drawn from a culturally homogeneous M–E bilingual population, groups at the higher and lower ends of the L2 proficiency spectrum were compared to test the possible effect of L2 proficiency in a clear way. In this case, L2 proficiency was used as a categorical variable, and the chi-square test was used.

## 3 Results

### 3.1 Characteristics of participants and effect of L1 proficiency

The participants' language backgrounds were very similar. No participants reported higher L2 proficiency than L1 proficiency. 7.7% of participants reported using such L3s as Hmong (*n* = 9), a language used by an ethnic minority group in China, Japanese (*n* = 4), and Korean (*n* = 3), in addition to Mandarin and English. Participants gave high percentages of correct answers to Time Questions 1 and 2, indicating that participants could understand the task.

For the target question, Time Question 3, there were generally more *Earlier/Past* interpretations. 80.7% of the participants (*n* = 292) gave the *Earlier/Past* interpretation, regardless of the testing language condition they were in. Logistic regression showed that L1 proficiency did not predict the participants' interpretation (β = 0.205, *p* = 0.287). Furthermore, participants were more likely to give the *Past/Earlier* interpretation when tested in L1 than when tested in L2, χ^2^ (1, *N* = 362) = 15.064, *p* < 0.01. Specifically, in the L1 testing condition, 88.6% of participants (*n* = 163) gave the *Past/Earlier* interpretation. In the L2 testing condition, 72.5% of participants (*n* = 129) gave the *Past/Earlier* interpretation.

### 3.2 Effect of L2 proficiency

L2 proficiency (i.e., L2 proficiency OBJ) was first used as a scale variable, and a binary logistic regression test showed that L2 proficiency did not predict the way participants interpreted *qián*-*forward* (β < 0.001, *p* = 0.851).

To maximize the effect of L2 proficiency in this sample, L2 proficiency was treated as a categorical variable. Participants whose L2 proficiency OBJ was higher than or equal to 528 (i.e., higher than the mean of 492.9 points + half standard deviation of 34.947 points) were classified into the higher L2 proficiency group (*n* = 116, 97 females; *M*_L1Proficiency_ = 3.047, *SD*_L1Proficiency_ = 0.685; *M*_L2Proficiency_ = 572.98, *SD*_L2Proficiency_ = 27.594; *M*_age_ = 18.91, *SD*_age_ = 0.680). Participants whose L2 proficiency OBJ was lower than or equal to 457 (i.e., lower than the mean of 492.9 points – half standard deviation of 34.947 points) were classified into the lower L2 proficiency group (*n* = 123, 77 females; *M*_L1Proficiency_ = 3.955, *SD*_L1Proficiency_ = 0.73; *M*_L2Proficiency_ = 413.5, *SD*_L2Proficiency_ = 29.939; *M*_age_ = 19.08, *SD*_age_ = 0.697). It was shown that the L2 proficiency group was not related to the participants' interpretation, χ^2^ (1, *N* = 239) = 0.11, *p* = 0.523, the result of which was consistent with the logistic regression result when L2 proficiency was used as a scale variable.

To test Hypothesis 2, further comparisons were made between the lower and higher L2 proficiency groups. As shown in Table 4, participants in the lower L2 proficiency group responded similarly in the two language conditions, χ^2^ (1, *N* = 123) = 0.169, *p* = 0.428. Specifically, 80.2% of participants in the L1 condition gave the *Past/Earlier* interpretation, and 79% of participants in the L2 condition gave the same interpretation. In contrast, participants in the higher L2 proficiency group responded differently when different testing languages were used, χ^2^ (1, *N* = 116) = 13.571, *p* < 0.001. Participants in this group gave the *Past/Earlier* interpretation more frequently in the L1 condition than in the L2 condition (93.5 vs. 66.7%), and they gave the *Future/Later* interpretation more frequently in the L2 condition than in the L1 condition (33.3 vs. 6.5%).

**Table 4:** *Past/Early* interpretations from the lower and higher L2 proficiency groups.

	**L1 condition**	**L2 condition**	***p*-value**
Percentage of participants who gave the *Past/Early* interpretation in the lower L2 group	82% (*n* = 61)	79% (*n* = 62)	0.428
Percentage of participants who gave the *Past/Early* interpretation in the higher L2 group	93.5% (*n* = 62 )	66.7% (*n* = 54 )	< 0.001

## 4 Discussion

Using a sample of M–E bilingual college students, this study investigates how L1 and L2 proficiencies would affect the retrieval of temporal concepts. We found that L1 temporal concepts would be retrieved more frequently than L2 temporal concepts, regardless of the testing languages. Furthermore, L1 and L2 proficiencies were not predictors of the way of interpretation (i.e., the way of retrieving L1 or L2 temporal concepts). L2 proficiency interacts with the testing languages on bilinguals' responses. Specifically, the higher L2 proficiency group could retrieve temporal concepts in line with the testing languages with higher accuracy than the lower L2 proficiency group. The study may shed some light on the temporal concept representation of this group of bilingual young adults who have lived in Mainland China since birth, and it may have added to the literature on the impact of language proficiency on concept representation and retrieval.

### 4.1 L1 temporal concepts were retrieved more frequently than L2 temporal concepts in both language conditions

Regardless of the testing languages, L1 temporal concepts were more retrievable for this group of M–E bilinguals, confirming Hypothesis 1a and what many lexicon models predict. According to lexicon models describing bilinguals' unbalanced L1 and L2 proficiencies, bilinguals' L1 lexicon would be larger than the L2 lexicon (e.g., Potter et al., 1984; Kroll and Stewart, 1994; Dong et al., 2005; Pavlenko, 2009), and/or the access links between L1 lexical items and L1 concepts would be stronger (e.g., Kroll and Stewart, 1994; Dong et al., 2005; Li, 2017), and/or the L1 concept inventory would be larger than the L2 concept inventory (e.g., Pavlenko, 2009; Li, 2017; Li, 2019). The result shows that the M–E bilingual college students living in Mainland China are bilinguals with higher L1 proficiency and lower L2 proficiency. The result is also consistent with that of Li and Zhang (2019). In Li and Zhang's (2019) study, 58% of M–E bilinguals who were postgraduates majoring in English retrieved L1 temporal concepts in the L1 condition, and 53% of M–E bilinguals with similar backgrounds retrieved L1 temporal concepts in the L2 condition.

In addition, L1 temporal concepts were retrieved more frequently in the present studies than in Li and Zhang's (2019) and Lai and Boroditsky's (2013) study. In Li and Zhang's (2019) study, although L1 temporal concepts were also retrieved more frequently than L2 temporal concepts in the two testing conditions, the retrieval frequencies of L1 temporal concepts were lower than those in the present study. In this study, 88.6% of participants in the L1 condition and 72.5% of participants in the L2 condition retrieved L1 temporal concepts. In Lai and Boroditsky's (2013) study, M–E bilingual residing in the United States were recruited, and 59% of participants in the L1 condition retrieved L1 temporal concepts by giving the *Past/Earlier* interpretation (c.f., 88.6% of participants gave the same response in the same condition in our study). The result would suggest the constraint of L2 learning contexts on L2 conceptualization. Kroll and Tokowicz (2005, p. 542) suggested that the learning context of bilinguals would have an impact on bilinguals' conceptualization. Studies have shown the importance of context in EFL learning (Herrington et al., 2003; Yang, 2006; Wong, 2013; Blyth, 2018; Lee and Park, 2019). The L2 learning contexts were limited because most of the participants in our study learn L2 in an environment of native L1 speakers, and their L2 immersion was very limited. The limited L2 learning context and immersion may limit their formation of L2 concepts.

### 4.2 L1 and L2 proficiencies did not predict the way of interpretation

Different from what was hypothesized in Hypothesis 1b, L1 and L2 proficiencies were not predictable for the way of interpretation. This result was different from that of Lai and Boroditsky (2013). In their study, L1 proficiency was a predictor for the retrieval of L1 temporal concepts, and L2 proficiency was a predictor for the retrieval of L2 temporal concepts. The difference in results may be explained by the fact that the demographic and language backgrounds of the sample in our study were much more homogeneous than those in their study. The participants in our study were from the same university, whereas their study recruited native English speakers residing in the United States and native Mandarin speakers residing in Taiwan. In our study, the L1 proficiency spectrum and the L2 proficiency spectrum of the participants may not be large enough, so that L1 and L2 proficiencies did not predict which language's temporal concepts would be retrieved.

### 4.3 Higher L2 proficiency means better retrieval in line with the testing languages

The result confirms Hypothesis 2. This result may be explained by the combination of different lexicon models. Concepts may be represented and accessed differently for bilinguals with different L2 proficiency, as described by different lexicon models. Specifically, when L2 proficiency was at the preliminary level, L2 concepts would be represented and accessed with reliance on L1 concepts and lexical items, as predicted by the Word Association Model (Potter et al., 1984). The Concept Mediation Model (Potter et al., 1984) and the Revised Hierarchical Model (Kroll and Stewart, 1994) can account for the intermediate L2 proficiency situation where L1 and L2 temporal concepts may be represented together so that L1 could access L2 temporal concept and L2 could access L1 temporal concept. When L2 proficiency was at the advanced level, concepts would be represented with language tags, as predicted by the models of Dong et al. (2005), Pavlenko (2009), Li (2017), and Li (2019), so that L1 could access L1 temporal concepts with higher accuracy. There may not be a clear cut between different L2 proficiency levels, but there may be characteristics. The participants in our study may be at different points along the L2 proficiency continuum, with some showing more characteristics of intermediate L2 bilinguals and others showing more characteristics of advanced L2 bilinguals.

This result may also suggest that bilinguals with higher L2 proficiency would have an attentional and/or inhibitory control advantage, so that they could more accurately retrieve L1 temporal concepts and inhibit L2 temporal concepts in the L1 condition, and retrieve L2 temporal concepts and inhibit L1 transfer in the L2 condition. The result may indicate the role of the SAS in bilinguals' mental processing. The SAS has been proposed to regulate the bilingual lexical-semantic system (Shallice and Burgess, 1996; Green, 1998). Since bilinguals have to deal with many different cognitive control processes (e.g., monitoring conflict suppressing interference, detecting cues, etc.) in the interactional context (Green and Abutalebi, 2013), the use of two languages provide cognitive benefits to bilinguals (e.g., Diamond, 2010). The cognitive benefits of bilingualism have been supported by studies (e.g., Hilchey and Klein, 2011; Bialystok and Craik, 2022; Xie et al., 2023). In addition, studies have supported a positive relationship between M–E bilinguals' L2 proficiency and inhibitory control (Privitera et al., 2022), and between L2 proficiency and attentional control (Privitera et al., 2023). Li and Zhang's (2019) study showed that M–E bilinguals' retrieval of temporal concepts was closely related to the cognitive load of the task. When cognitive resources were allocated to the difficult task, it was difficult for participants (even those with high L2 proficiency) to inhibit L1 transfer when tested in L2 (Li and Zhang, 2019). In our study, bilinguals with higher L2 proficiency were better able to give interpretations in line with the testing languages, suggesting that there may be cognitive benefits to bilingualism.

## 5 Limitation

As the participants in our study were homogeneous in terms of demographics and education, dimensions of language experience other than L1 and L2 proficiencies, such as AoA, L2 dominance, L1–L2 dominance ratio, and L2 immersion, were not included. Previous studies have shown that different dimensions of language experience would have different influences on bilinguals' cognitive processing (Gullifer et al., 2021; Privitera et al., 2023), so further studies may be conducted to examine the effects of multidimensional language experience.

We offered two explanations for the finding that the higher L2 proficiency group was more accurate in retrieving temporal concepts in line with the testing languages. On the one hand, different lexicon models might be used by bilinguals with different L2 proficiencies. On the other hand, it was possible that higher L2 proficiency was associated with cognitive advantages. This study could not determine which explanation would better explain the result. Further research may be conducted in this aspect.

Individual differences were not taken into account in our study. Previous studies have shown that pressure may influence adolescent Mandarin speakers' interpretation of the Mandarin temporal metaphor of *qián* (Li, 2015); and that power, procrastination, and cultural background would influence English native speakers' interpretation of the English temporal metaphor of *forward* (Duffy and Feist, 2014, 2017; Duffy et al., 2014; Li and Zhang, 2017). The following studies can examine whether individual differences interact with language experience on bilinguals' interpretations of spatio-temporal metaphors.

## 6 Conclusion

By investigating M–E bilingual young adults' interpretation of *qián*-*forward*, this study has examined the effects of L1 and L2 proficiencies on the retrieval of temporal concepts. It was found that L1 temporal concepts would be retrieved more frequently than L2 temporal concepts regardless of the testing languages, that L1 and L2 proficiencies were not predictors for the way of interpretation, and that the higher L2 proficiency group could retrieve temporal concepts in line with the testing languages with higher accuracy than the lower L2 proficiency group. The results suggest that bilinguals with higher L2 proficiency may be able to represent temporal concepts with language tags or have an attentional and/or inhibitory control advantage.

## Data availability statement

The raw data supporting the conclusions of this article will be made available by the authors, without undue reservation.

## Ethics statement

The studies involving humans were approved by the Scientific Research Ethics Committee of Hunan Normal University, China. The studies were conducted in accordance with the local legislation and institutional requirements. The participants provided their written informed consent to participate in this study.

## Author contributions

TY: Writing – original draft, Writing – review & editing. YY: Writing – review & editing.

## Footnotes

^1^There are differences for L2 AoA since some families would enroll kids in pre-school English classes, some primary schools with a foreign language focus offer English classes to 6- or 7-year-old students and other primary schools may offer English classes when students are 10 or 11 years old.

^2^The data are given on the website of the Ministry of Education of the People's Republic of China: http://www.moe.gov.cn/jyb_sjzl/moe_560/2021/quanguo/202301/t20230103_1037969.html.
